# Phagocytosis: A Fundamental Process in Immunity

**DOI:** 10.1155/2017/9042851

**Published:** 2017-06-12

**Authors:** Carlos Rosales, Eileen Uribe-Querol

**Affiliations:** ^1^Departamento de Inmunología, Instituto de Investigaciones Biomédicas, Universidad Nacional Autónoma de México, 04510 Ciudad de México, Mexico; ^2^División de Estudios de Posgrado e Investigación, Facultad de Odontología, Universidad Nacional Autónoma de México, 04510 Ciudad de México, Mexico

## Abstract

One hundred years have passed since the death of Élie Metchnikoff (1845–1916). He was the first to observe the uptake of particles by cells and realized the importance of this process for the host response to injury and infection. He also was a strong advocate of the role of phagocytosis in cellular immunity, and with this he gave us the basis for our modern understanding of inflammation and the innate and acquired immune responses. Phagocytosis is an elegant but complex process for the ingestion and elimination of pathogens, but it is also important for the elimination of apoptotic cells and hence fundamental for tissue homeostasis. Phagocytosis can be divided into four main steps: (i) recognition of the target particle, (ii) signaling to activate the internalization machinery, (iii) phagosome formation, and (iv) phagolysosome maturation. In recent years, the use of new tools of molecular biology and microscopy has provided new insights into the cellular mechanisms of phagocytosis. In this review, we present a general view of our current knowledge on phagocytosis. We emphasize novel molecular findings, particularly on phagosome formation and maturation, and discuss aspects that remain incompletely understood.

## 1. Introduction

Élie Metchnikoff (1845–1916) made his original observations in the 1880s while studying invertebrate marine organisms. He found special cells attacking small thorns placed into starfish larvae. Based on these findings, he later moved into immunology and championed the concept of cellular immunity. For his contributions he was awarded the Nobel Prize in 1908 [1]. He shared the prize with Paul Ehrlich, a supporter of humoral immunity. Together they provided the bases for modern immunology.

Phagocytosis is an important process for nutrition in unicellular organisms, while in multicellular organisms it is found in specialized cells called phagocytes. Phagocytosis consists in recognition and ingestion of particles larger than 0.5 *μ*m into a plasma membrane derived vesicle, known as phagosome. Phagocytes can ingest microbial pathogens, but importantly also apoptotic cells. In this way, they contribute to the clearance of billions of cells that are turned over every day. Thus phagocytosis becomes essential not only for microbial elimination, but also for tissue homeostasis. Professional phagocytes [2] include monocytes, macrophages, neutrophils, dendritic cells, osteoclasts, and eosinophils. These cells are in charge of eliminating microorganisms and of presenting them to cells of the adaptive immune system. In addition, fibroblasts, epithelial cells, and endothelial cells can also perform phagocytosis. These nonprofessional phagocytes cannot ingest microorganisms but are important in eliminating apoptotic bodies [3, 4].

Phagocytes must recognize a large number of different particles that could potentially be ingested, including all sorts of pathogens and also apoptotic cells. This recognition is achieved thanks to a variety of discrete receptors that distinguish the particle as a target and then initiate a signaling cascade that promotes phagocytosis. Receptors on the plasma membrane of phagocytes can be divided into nonopsonic or opsonic receptors. Nonopsonic receptors can recognize directly molecular groups on the surface of the phagocytic targets. Among these receptors there are lectin-like recognition molecules, such as CD169 and CD33; also related C-type lectins, such as Dectin-2, Mincle, or DNGR-1; scavenger receptors [5]; and Dectin-1, which is a receptor for fungal beta-glucan [6]. Other receptors, such as SR-A or CD36, can recognize both apoptotic and microbial polyanionic ligands, but their signaling capacity is not well described [5]. Interestingly, toll-like receptors (TLRs) [7] are detectors for foreign particles, but they do not function as phagocytic receptors. However, TLRs often collaborate with other nonopsonic receptors to stimulate ingestion [8].

Opsonic receptors recognize host-derived opsonins that bind to foreign particles and target them for ingestion. Opsonins include antibodies, complement, fibronectin, mannose-binding lectin, and milk fat globulin (lactadherin) [3]. The best characterized and maybe most important opsonic phagocytic receptors are the Fc receptors (FcR) and the complement receptors (CR). FcRs bind to the constant (Fc portion) of immunoglobulin (Ig) G [9, 10] or IgA antibodies [11]. Complement receptors, such as CR3, bind to iC3b deposited on the particle after complement activation [12].

After recognition of the target particle, phagocytic receptors initiate signaling cascades that remodel lipids in the cell membrane and regulate the actin cytoskeleton in order to extend the cell membrane around the particle [13]. During this part of the process, phagocytic receptors also engage in a sequential order and cooperate to complete the formation of the phagosome [14].

Once the particle is internalized inside the early phagosome, this vacuole can fuse with vesicles coming from the endoplasmic reticulum and the Golgi complex to form an intermediary phagosome [15–21]. The contribution of the endoplasmic reticulum to phagosome formation and maturation is not completely understood, particularly in relation to cross-presentation of antigens. This is the process by which MHC class I (MHC-I) molecules can also present peptides from extracellular proteins. MHC-I molecules are delivered to the phagosome, where they are loaded with peptide and then recycled back to the plasma membrane. At present, it is not possible to convincingly describe a trafficking pathway for MHC-I molecules leading to cross-presentation. While classic (endogenous) MHC-I loading is basically restricted to the secretory pathway, cross-presentation involves interaction between this pathway and the phagocytic pathway [22]. A complete discussion of cross-presentation is beyond the scope of the present review. The reader is directed to recent excellent reviews on this topic [23, 24]. Similarly, the contribution of the Golgi complex to phagosome formation is a matter of debate. Despite the fact that a role for the Golgi complex during phagocytosis by macrophages has been ruled out consensually by several groups [25–27], it is important to notice that these reports are mainly focused on Fc*γ* receptor-mediated phagocytosis. In contrast, it was recently reported that recruitment of Golgi-derived secretory vesicles during phagosome formation was important for uptake of most particles, except IgG-opsonized ones [20]. The formation of an intermediary phagosome is dynamic process involving fusion of endocytic vesicles and fission of secretory vesicles, resulting in remodeling of the membrane and progressive acidification of the phagosome [28]. Later this intermediary phagosome turns into a microbicidal vacuole, the phagolysosome, by fusing with lysosomes and changing its membrane and interior characteristics through a process named phagolysosome maturation [28].

## 2. Particle Recognition

The first step in phagocytosis is the detection of the particle by phagocytes. This, as mentioned before, is accomplished by specialized receptors on the cell membrane. Foreign particles, such as microbial pathogens, can be recognized directly by receptors that bind molecules not found in higher organisms, or indirectly through opsonins. Several receptor types are found on a single phagocyte and they cooperate for recognition and ingestion of the particle. Some receptors can bind to pathogen-associated molecular patterns (PAMPs) but not necessarily initiate phagocytosis. TLRs and some G-protein coupled receptors prepare (prime) the cell for phagocytosis by inducing inside-out activation of phagocytic integrins.

### 2.1. Receptors for Foreign Particles

#### 2.1.1. Pattern-Recognition Receptors

Some receptors that directly bind PAMPs and seem to be phagocytic receptors include Dectin-1, mannose receptors, CD14, and scavenger receptor A (SR-A) (Table 1). Dectin-1 binds to polysaccharides of some yeast cells [29]. Mannose receptors bind mannan [30]. CD14 binds to lipopolysaccharide-binding protein [31]. SR-A can detect lipopolysaccharide (LPS) on some gram-negative bacteria [32] and on* Neisseria meningitidis* [33]. Among these receptors, Dectin-1 has been clearly shown to be sufficient for activating phagocytosis. When it is expressed on heterologous cells that normally cannot perform phagocytosis, it gives the cells phagocytic capabilities [29, 34]. However, for other PAMP receptors the phagocytic potential is still a matter of debate. It may be that they induce phagocytosis indirectly by tethering the particle to the phagocyte surface, or by priming the phagocyte [35] to ingest the particle via other receptors.

#### 2.1.2. Opsonic Receptors

Foreign particles can also be recognized by phagocytes through soluble molecules that will bind to the particles, tagging them for ingestion. Once on the surface of the target particle, these molecules, called opsonins, are in turn recognized by specific receptors on the membrane of phagocytes. In this manner, opsonins function as a bridge between the phagocyte and the particle to be ingested. Antibody (IgG) molecules and complement components are important opsonins that induce efficient phagocytosis, and their receptors have been studied extensively (Table 1). Fc*γ* receptors (Fc*γ*R) are a family of glycoproteins expressed on the membrane of leukocytes, capable of binding the Fc portion of IgG molecules [10, 36]. These receptors can bind to the various IgG subclasses with different affinities [9] and when crosslinked by multivalent antigen-antibody complexes can induce phagocytosis and other cellular responses [9]. Complement receptors (CRs) recognize components of the complement cascade, deposited on the surface of phagocytic targets [37]. There are now three recognized gene superfamilies of complement receptors: (i) the short consensus repeat (SCR) modules that code for CR1 and CR2, (ii) the *β*2 integrin family members CR3 and CR4, and (iii) the immunoglobulin Ig-superfamily member CRIg [12]. Complement receptors, such as the integrin *α*_M_*β*2 (also known as CD11b/CD18, CR3, or Mac-1), bind the complement component iC3b deposited on pathogens to promote phagocytosis [38, 39].

### 2.2. Receptors for Apoptotic Cells

In addition to foreign pathogens, in a normal organism there are millions of cells that die by apoptosis every day. These apoptotic bodies are constantly cleared by phagocytosis. Recognition of apoptotic bodies involves several signals. First, cells in apoptosis release molecules that normally do not exist outside cells. Some of these molecules include ATP, lysophosphatidylcholine, and sphingosine 1-phosphate. These soluble molecules function as chemoattractants for phagocytes. Also, apoptotic cells are displayed on their surface molecules, such as phosphatidylserine (PS) not normally present on a healthy cell [54]. These surface molecules function as an “eat me” signal [55] for phagocytes. Some receptors such as TIM-1, TIM-4 [48], stabilin-2 [49], and BAI-1 (brain-specific angiogenesis inhibitor 1) directly recognize PS [50]. Other receptors, for example, MFG-E8 (lactadherin), can connect PS to *α*V*β*3 integrins [51]. Apoptotic cells can also be recognized by scavenger receptors A (SR-A), MARCO, and CD36 [56]. CD36 bind modified lipids, including oxidized PS [53]. Many normal cells can also express some amounts of PS on their membranes. However, PS increases as much as 300-fold in apoptotic cells, creating a threshold that prevents phagocytosis of normal cells. There are some cells, for example, activated B and T cells, that may present large amounts of PS on their membrane. To prevent phagocytosis, these cells express molecules that deliver a “do not eat me” signal [4]. CD31 is one such molecule. It prevents phagocytosis by promoting cell detachment after homotypic (self)-binding [57]. Also, CD47 is another molecule that blocks phagocytosis of cells expressing it on their surface. CD47 binds to the receptor SIRP*α* (signal regulatory protein *α*), on the membrane of phagocytes, and delivers an inhibitory signal for actin assembly [58]. Another level of complexity is the fact that multiple receptors bind apoptotic cells directly or indirectly and professional phagocytes coexpress many of these receptors. Thus, there are still many unidentified mechanisms for phagocytosis via apoptotic receptors. Because, it is now recognized that clearance of apoptotic cells is fundamental for tissue homeostasis [59], future research will bring us great surprises in this area.

### 2.3. Receptor Cooperation

For an efficient recognition of the target particle, multiple receptors on the phagocyte must engage multiple ligands on the particle. This interaction depends on the relative affinity of the molecules involved and also on their density on the surface of both the leukocyte and the particle. In addition, the relative mobility of the receptors on the membrane of the phagocyte affects the avidity of the interaction [60]. Because phagocytic receptors get activated when they aggregate in the plane of the membrane, only receptors capable of fast lateral diffusion are more likely to form multimers and get activated than immobile receptors (see section on phagosome formation). Aggregation (also called crosslinking) of the receptors is additionally promoted by the active nature of phagocytes, which constantly form membranous projections to probe their environments [61, 62]. Thus, particle recognition by receptor binding and activation are very active processes.

Another aspect of receptor cooperation is observed when integrin receptors, such as the CR3, increase their affinity for their ligand only after the phagocyte gets extra stimuli through TLRs [63], Fc receptors [64], or CD44 [65]. These receptors initiate intracellular signaling that activates the small GTPase Rap1 [66], which in turn provokes conformational changes in the integrin, leading to its increased affinity. This process is called inside-out signaling because the signal that activates the integrin comes from inside the cell. During the phagocytic process integrins get activated to promote efficient receptor binding all around the target particle (see later).

## 3. Particle Internalization

When a particle interacts with phagocyte receptors, a series of signaling events are triggered to activate phagocytosis. Important changes in membrane remodeling and the actin cytoskeleton take place leading to the formation of pseudopods that cover the particle. At the point of contact, a depression of the membrane (the phagocytic cup) is formed. Then, the membrane surrounds the target particle and within few minutes it closes at the distal end, leaving a new phagosome. The signaling cascades are known in great detail for the Fc receptors and the complement receptors, since these are the best-studied phagocytic receptors [38, 67, 68]. Signaling for other phagocytic receptors is just beginning to be explored. Great interest exists in this area and research will certainly be fruitful in the near future.

### 3.1. Fc*γ* Receptor Signaling

Fc*γ* receptors get activated in the plane of the phagocyte membrane when they aggregate after binding to their IgG ligands that cover the particle to be ingested. In humans there are several types of activating Fc*γ*Rs that are coexpressed by professional phagocytes along with the only inhibitory Fc*γ*RIIb. The clustering of activating Fc*γ*Rs results in the phosphorylation of immunoreceptor tyrosine-based activation (ITAM) motifs present within the cytoplasmic domain of the receptor (as is the case with Fc*γ*RIIa and Fc*γ*RIIc), or in an associated FcR common *γ*-chain (as with Fc*γ*RI and Fc*γ*RIIIa) [9, 10, 69]. ITAM phosphorylation is carried out by Src-family kinases (Lyn, Lck, and Hck specifically), creating a docking site for the SH2 domains of the tyrosine kinase Syk, which can itself phosphorylate neighboring ITAM tyrosines [38, 70]. The mechanism by which receptor aggregation induces phosphorylation of the ITAM tyrosines remains elusive. Aggregation may induce accumulation of the Fc*γ*Rs in cholesterol-enriched lipid rafts, where Src-family kinases are concentrated. This model is supported by the fact that Fc*γ*RIIa becomes associated with detergent-resistant membranes (DRMs) upon activation by aggregation [71, 72] and that depletion of cholesterol with methyl-*β*-cyclodextrin inhibits Fc*γ*RII phosphorylation in response to aggregation [71]. Association of Fc*γ*RIIa with DRMs depends on its palmitoylation on a cysteine residue [73]. Despite these reports, the model of lipid rafts presents some limitations that need to be considered. For example, not all Fc*γ* receptors are palmitoylated (like Fc*γ*RIIa is); thus other receptors may not associate with lipid rafts or they would do by another mechanism. Interestingly, a transmembrane mutant form of Fc*γ*RIIa that failed to associate with lipid rafts was still able to trigger phagocytosis [73]. Also, the use of methyl-*β*-cyclodextrin to eliminate cholesterol from the cell membrane may be a very harsh treatment and the functional condition of the cell afterwards is not clear. Moreover, lipid rafts disruption by cholesterol depletion did not inhibit phagocytosis in macrophages [74]. In addition, there is still a debate whether DRMs really reflect the segregation of lipids in membranes or are artificially induced by the detergents used in their preparation. Thus, the model of lipid rafts needs to be considered with caution [75].

As mentioned above, different phagocytes express more than one activating Fc*γ*R, and at the same time they also express the inhibitory Fc*γ*RIIb. The coexpression of both activating and inhibitory Fc*γ*R results in simultaneous triggering of activating and inhibitory signaling pathways [10]. Thus, a particular phagocyte will initiate phagocytosis when the sum of activating and inhibiting signals reaches a threshold of activation that is determined by the relative expression of both types of Fc*γ*R [76]. The importance of the inhibitory Fc*γ*RIIb in regulating many IgG-mediated responses in different leukocytes was made evident in Fc*γ*RIIb-deficient mice, which showed enhanced activity of many IgG-mediated cell responses including phagocytosis [77]. Another molecule that negatively regulates phagocytosis of macrophages is CD47 via SIRP*α* [78, 79]. Ligation of CD47 leads to phosphorylation of the immunoreceptor tyrosine-based inhibition (ITIM) motif in the cytoplasmic tail of SIRP*α*, which in turn recruits the phosphatase SHP-1 [58]. By super-resolution microscopy, it has become evident that many receptors are found in clusters at the plasma membrane on a nanometer scale [80]. In the case of resting macrophages, it was recently found that nanoclusters of Fc***γ*** RI are constitutively associated with nanoclusters of SIRP*α*. Upon Fc receptor activation, Src-family kinase signaling leads to segregation of Fc***γ***RI and SIRP*α* nanoclusters [81], and co-ligation of SIRP*α* with CD47 prevented nanocluster segregation. Thus, when the balance of signals favors activation, Fc*γ*RI nanoclusters are separated from the inhibitory signal [81].

**Figure 1:**
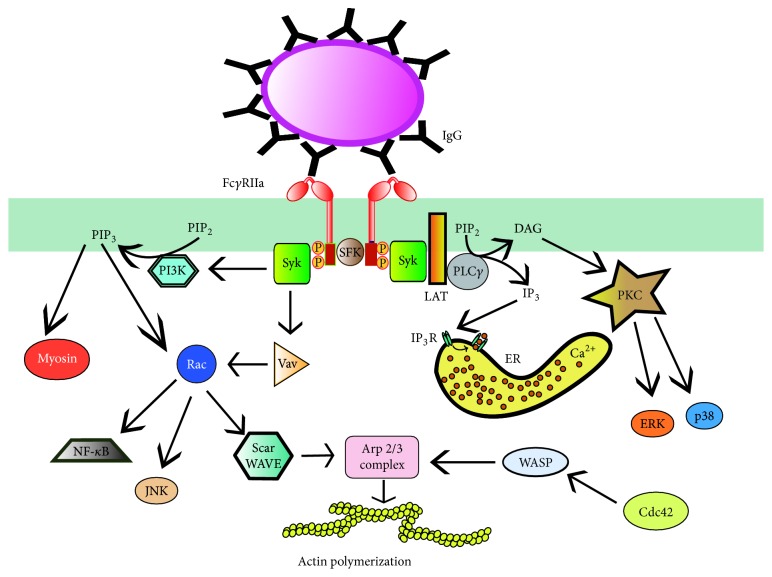
*Fcγ receptor signal transduction*. Fc*γ*RIIa crosslinking by immunoglobulin (IgG) bound to a particle induces activation of Src family kinases (SFK), which phosphorylate tyrosine residues in the ITAMs (red box) of the cytoplasmic tail of the receptor. Then, Syk associates with phosphorylated ITAMs and leads to phosphorylation and activation of a signaling complex formed by the scaffold protein LAT (linker for activation of T cells) interacting with various proteins. Some of these proteins are phospholipase C gamma (PLC*γ*), which produces inositoltrisphosphate (IP_3_) and diacylglycerol (DAG). These second messengers cause calcium release and activation of protein kinase C (PKC), respectively. PKC leads to activation of extracellular signal-regulated kinases (ERK and p38). The guanine nucleotide exchange factor Vav activates the GTPase Rac, which is involved in regulation of the actin nucleation complex Arp2/3, via the nucleation-promoting factor Scar/WAVE. Rac is also involved in activation of transcription factors such as NF- *κ*B and JNK. The enzyme phosphatidylinositol 3-kinase (PI3K), which is recruited and activated by Syk, generates the lipid phosphatidylinositol-3,4,5-trisphosphate (PIP_3_) at the phagocytic cup. This lipid also regulates Rac activation and contractile proteins such as myosin. Another GTPase, Cdc42, is also activated during Fc*γ*R signaling by an unknown mechanism and induces actin polymerization by activating the nucleation-promoting factor WASp (Wiskott-Aldrich Syndrome protein). P represents a phosphate group. ER, endoplasmic reticulum.

After Fc*γ*R phosphorylation, Syk binds to the ITAM motifs and gets also activated. Syk has also been shown to be required for phagocytosis [38, 70] and it is responsible for activation of several additional signaling proteins that get recruited to the Fc*γ*R signaling complex (Figure 1). The transmembrane protein LAT (linker for activation of T cells) is phosphorylated by Syk. Phosphorylation of LAT induces docking of additional adaptors: Grb2 binds to LAT, and in turn it recruits Gab2 (Grb2-associated binder 2). Gab2 is also phosphorylated by Syk. Other proteins are then also recruited to the complex. Among them is phospholipase C (PLC) *γ*1, which produces inositoltrisphosphate (IP_3_) and diacylglycerol (DAG). These second messengers cause calcium release and activation of protein kinase C (PKC), respectively. PKC leads to activation of extracellular signal-regulated kinases (ERK and p38) [82]. The guanine nucleotide exchange factor (GEF) Vav activates GTPases of the Rho and Rac family, which are involved in regulation of the actin nucleation complex Arp2/3, which induces the actin polymerization that drives pseudopod extension. Other enzymes such as phosphatidylinositol 3-kinase (PI 3-K) activate the GTPase Rac and nuclear factors like NF-*κ*B (Figure 1).

#### 3.1.1. Lipid Signals

**Figure 2:**
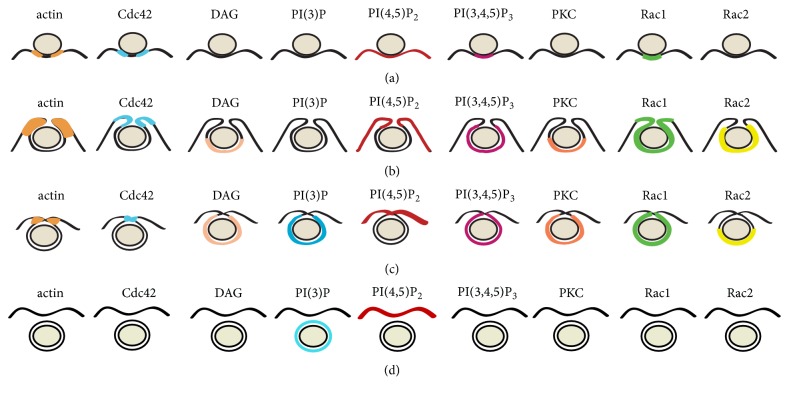
*Signaling molecules concentrated in different parts of the membrane during phagocytosis*. A phagocyte cell membrane around an IgG-opsonized particle is shown at different stages of phagosome formation. After initial recognition, membrane protrusions form a phagocytic cup (a), then pseudopods extend around the particle (b), and membrane fusion events at the distal end close the new vacuole (c), which is finally separated as an intracellular phagosome (d). Fluorescent protein chimeras were used to locate (colored lines) the signaling molecules PI(4,5)P2, DAG, PKC, PI(3,4,5)P3, PI(3)P, active (GTP-bound) Cdc42, Rac1, Rac2, and actin.

Signaling events regulating phagosome formation have also been examined by fluorescence imaging techniques. Detection of lipids and several activating proteins has shown that different molecules associate and dissociate from phagosomes in an orderly fashion (Figure 2). Phosphatidylinositol-4,5-bisphosphate [PI(4,5)P_2_] is present in large amounts in the inner leaflet of the plasma membrane of resting phagocytes. During phagocytosis, the concentration of PI(4,5)P_2_ increases in the pseudopods that form the phagocytic cup but then decreases abruptly [83]. The drastic disappearance of PI(4,5)P_2_ following its modest initial accumulation is essential to allow particle internalization, probably by facilitating actin disassembly [84]. Several pathways contribute to the disappearance of PI(4,5)P_2_. PLC*γ* is phosphorylated and recruited to the phagocytic cup in a Syk-dependent manner, probably by interaction with LAT [83, 85]. PLC*γ* activity is critical because its inhibition prevents DAG production and blocks phagocytosis [83]. In addition, DAG leads to activation of PKC*ε*, which enhances phagocytosis [86]. PI(4,5)P_2_ is also consumed when it becomes phosphorylated by PI-3K, producing PI(3,4,5)P_3_ at the phagocytic cup [87]. PI-3K is recruited and activated by Syk [88], or by adaptor proteins such as Gab2 [89] (Figure 1). These dramatic changes in membrane lipid composition during Fc*γ* receptor-mediated phagocytosis demonstrate that distinct molecules are activated and recruited in a carefully orchestrated manner to induce phagosome formation.

#### 3.1.2. Small GTPases

Small GTPases of the Rho family are important regulators of the actin cytoskeleton. These enzymes function as molecular switches alternating between an active (GTP-bound) state and an inactive (GDP-bound) state [90]. For activation, they need to release GDP and replace it with GTP. This action is catalyzed by guanine nucleotide exchange factors (GEFs). Later, GTP is hydrolyzed to GDP returning the GTPase to its inactive state. This last step is enhanced through interactions with GTPase-activating proteins (GAPs). The GTPases Rac and Cdc42 are activated and recruited to the forming phagosome during Fc*γ* receptor-mediated phagocytosis (Figure 1) [91]. Cdc42 is activated early in phagocytosis mostly at the rims of the phagocytic cup [92] (Figure 2). Rac1 is activated throughout the entire nascent phagosome, whereas Rac2 is activated later, mostly at the base of the phagocytic cup [92] (Figure 2). Cdc42 and Rac participate in regulating the localized formation of actin fibers, necessary for pseudopod extension, by activating the nucleation-promoting factors WASp (Wiskott-Aldrich Syndrome protein) and Scar/WAVE, respectively [93] (Figure 1). WASp and Scar, in turn, activate the Arp2/3 complex for actin polymerization [94] (Figure 1).

### 3.2. Complement Receptor Signaling

The integrin CR3 is the best-studied phagocytic complement receptor. For a long time, it has been recognized that engagement of CR3 on macrophages triggers a distinct form of phagocytosis, characterized by “sinking” of the particle into the cell without forming the characteristic pseudopods of Fc*γ*R phagocytosis [95]. However, this idea has been questioned by recent microscopy observations that showed membrane protrusions encircling the targets during CR3-mediated phagocytosis [62, 96]. Still, it is thought that integrin CR3 signaling for phagocytosis is very different from Fc*γ*R signaling. Early reports demonstrated that phagocytosis of complement- opsonized zymosan and of complement-opsonized erythrocytes was unaffected by tyrosine kinase inhibitors [97]. This ruled out the participation of tyrosine kinases in this type of phagocytosis. In addition, macrophages from Syk^−/−^ mice showed normal levels of CR-mediated phagocytosis [98]. However, *β*2 integrin stimulation by adhesive ligands, or by artificial integrin cross-linking with antibodies induced various cellular responses in a Src and/or Syk kinase-dependent manner [99]. More recently, it was shown that Syk is phosphorylated during CR3-mediated phagocytosis and its inhibition prevents particle ingestion [100]. Also, Syk can be indirectly activated by integrins via the ITAM-bearing FcR *γ* chain and/or DAP12 [101]. The reason Syk^−/−^ macrophages are capable of CR-mediated phagocytosis while the other experimental systems clearly implicate Syk in integrin signaling remains a mystery. It might be possible that genetically deficient cells have upregulated other molecules, for example, Zap70, that allow the bypass of Syk during CR-mediated phagocytosis.

Other differences between Fc*γ*R- and CR-mediated phagocytosis seem to be the cytoskeleton requirements for particle internalization. The actin cytoskeleton is required for Fc*γ*R-mediated phagocytosis, whereas the actin and microtubule cytoskeletons are required for CR-mediated phagocytosis [97, 102]. Moreover, in complement phagocytosis F-actin accumulation and particle ingestion depend on RhoA, but not on Rac or Cdc42 [103, 104], and binding of iC3b-opsonized erythrocytes increased levels of Rho-GTP but not of Rac-GTP [105]. However, ingestion of iC3b-opsonized erythrocytes is reduced in cells where Rac1 and Rac2 were deleted [106]. Together these findings challenge the classical model that CR3-mediated phagocytosis depends only on RhoA [106].

**Figure 3:**
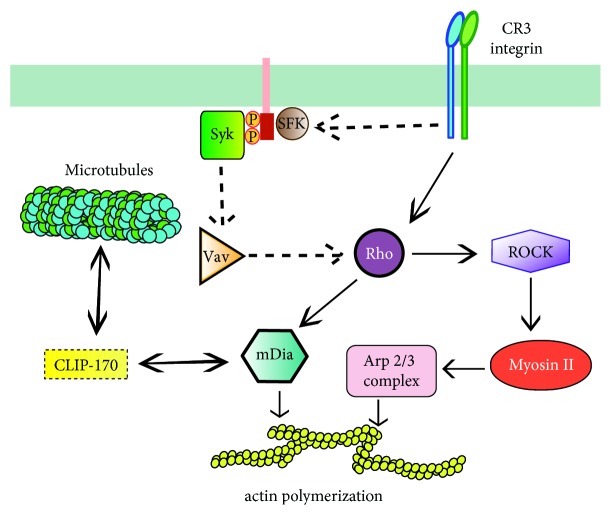
*Complement receptor signaling in phagocytosis*. The complement receptor 3 (CR3 integrin) binds the complement fragment iC3b and initiates a signaling cascade that activates Rho, either independently of tyrosine kinases (in macrophages) or via Syk, which is recruited through an ITAM-bearing molecule (such as DAP12 or the Fc receptor *γ* chain). Syk may also activate the GEF Vav to further activate Rho. Rho, in turn, leads to actin polymerization via two mechanisms. Rho can activate Rho kinase (ROCK), which phosphorylates and activates myosin II, inducing accumulation of Arp2/3 and actin assembly at the phagocytic cup. Rho can also induce accumulation of mDia1 (mammalian diaphanous-related formin 1), which promotes actin polymerization. In addition, mDia1 binds directly to the microtubule-associated protein CLIP-170 providing a link to the microtubule cytoskeleton. P represents a phosphate group. ITAM, immunoreceptor tyrosine-based activation motif.

Rho, in turn, leads to actin polymerization via two mechanisms (Figure 3). First, Rho can activate Rho kinase, which phosphorylates and activates myosin II [107]. Inhibition of Rho kinase activity also prevents accumulation of Arp2/3 and actin assembly at the phagocytic cup [107]. Second, Rho can induce accumulation of mDia1 (mammalian diaphanous-related formin 1) and polymerized actin in the phagocytic cup. Interfering with mDia activity inhibits CR3-mediated phagocytosis while having no effect on Fc*γ*R-mediated phagocytosis [108]. Also, mDia1 binds directly to the microtubule-associated protein CLIP-170 and induces its accumulation at the phagocytic cup [109]. This pathway also provides a link to the microtubule cytoskeleton required for CR-mediated phagocytosis [97, 102]. Thus, microtubules and actin seem to function cooperatively in CR-mediated phagocytosis (Figure 3).

The signaling pathway for Rho activation is not clearly defined. Two regions in the cytosolic domain of the *β*2 subunit of the integrin receptor are important for Rho activation during phagocytosis [105], but it is not clear how the integrin connects to a Rho GEF for activation. In addition, Vav (a Rho/Rac GEF) originally reported to participate in Fc*γ*R-mediated phagocytosis, but not in CR-mediated phagocytosis [110], can also activate Rho [106]. Since, Rho participates in Arp2/3 activation and actin polymerization by CR3 [104] and Vav is a substrate for Syk [111], it is possible that a connection exists for Rho activation via Syk and Vav [3] (Figure 3).

## 4. Phagosome Formation

As indicated before, phagocytosis commences by interaction of phagocytic receptors with ligands on the surface of target particles. Then, receptors must aggregate to initiate signaling pathways that regulate the actin cytoskeleton, so that the phagocyte can produce membrane protrusions for involving the particle. Finally, the particle is enclosed in a new vesicle that pinches out from the plasma membrane.

### 4.1. Initial Interactions

**Figure 4:**
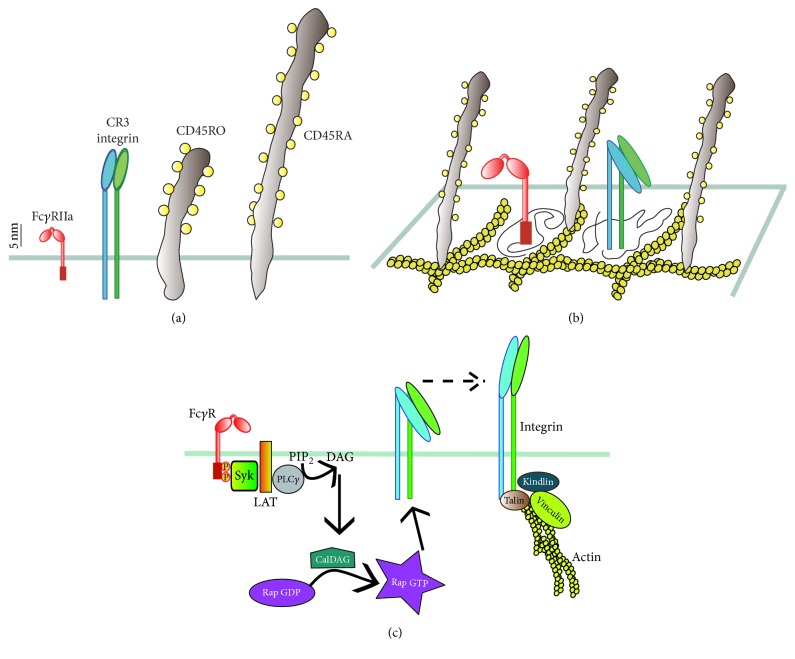
*Cooperation among phagocytic receptors*. (a) Most phagocytic receptors, such as receptors for antibody (Fc*γ*RIIa) and receptors for complement (Integrin CR3) are small molecules that extend only few nanometers from the plasma membrane. In contrast, transmembrane glycoproteins, such as phosphatases CD45 (CD45RO and CD45RA isoforms), are much longer and usually rigid molecules. (b) In the resting state, receptors cannot diffuse freely throughout the membrane. Their movement is restricted by fences of transmembrane glycoprotein “pickets” attached to an actin mesh. (c) Fc*γ*R aggregation triggers an inside-out signal that activates integrins. Fc*γ*R-induced activation of phospholipase C (PLC) produces diacylglycerol (DAG) that leads to activation of CalDAG (a Rap GEF), which in turn activates Rap. Activated Rap (Rap GTP) is responsible for integrin activation by disrupting interactions between integrin subunits and promoting binding to talin, vinculin, and the actin cytoskeleton.

The initial interactions of phagocytic receptors with the particle are not easy, since receptor ligands do not usually cover the particle uniformly and receptors are not freely accessible on the cell membrane. In fact, most phagocytic receptors are short molecules that extend only around 5 nm from the surface of the cell (Figure 4(a)) and are found among many much longer, usually rigid, transmembrane glycoproteins present throughout the membrane. These glycoproteins form a thick layer, known as glycocalyx, covering the cell membrane, that can effectively conceal short receptors [112]. Mucins, high molecular weight, heavily glycosylated proteins, CD44 and hyaluronan, and transmembrane phosphatases such as CD45 and CD148 are components of the glycocalyx that can reduce ligand access to receptors on the phagocyte membrane (Figure 4(a)). In addition, the lateral diffusion of receptors on the cell membrane can be effectively reduced by glycocalyx components that are tethered to cytoskeletal structures. These glycoproteins effectively act as the “pickets” of a cytoskeletal “fence” [13, 14] that impedes free diffusion of other membrane molecules. This is the case for phagocytic receptors, which move only in discrete areas on the cell membrane among these immobile picket fences (Figure 4(b)).

Phagocytes improve interactions of receptors with possible targets by (i) creating active membrane protrusions that allow the cell to explore larger areas, increasing the chances for receptors to engage their ligands [61, 113], and by (ii) selectively removing some of these larger glycoproteins allowing the receptors to diffuse more freely on the membrane [114]. The phosphatase CD45 can extend more than 40 nm from the cell membrane [115], and it is a real steric obstacle for phagocytic receptors. Removing these large molecules could greatly improve receptor binding. Indeed, removal of CD45 was first observed during Dectin-1-mediated phagocytosis in a structure that was called “phagocytic synapse” [116], for its similarity to the T lymphocyte immune synapse [117]. When T cell receptor (TCR) molecules on the T lymphocyte interact with MHC/peptide molecules on an antigen-presenting cell, a central cluster of engaged TCR is formed. The TCRs are surrounded by a ring of integrin LFA-1 (lymphocyte-function-associated antigen-1) molecules, and CD45 is excluded from the central area. TCR interactions span around 15 nm, while integrin interactions span around 30–40 nm between the two cells. Thus removal of the larger molecules helps an efficient TCR interaction. A similar situation for Fc*γ*R-mediated phagocytosis has also been elegantly described recently by Sergio Grinstein's group [114].

**Figure 5:**
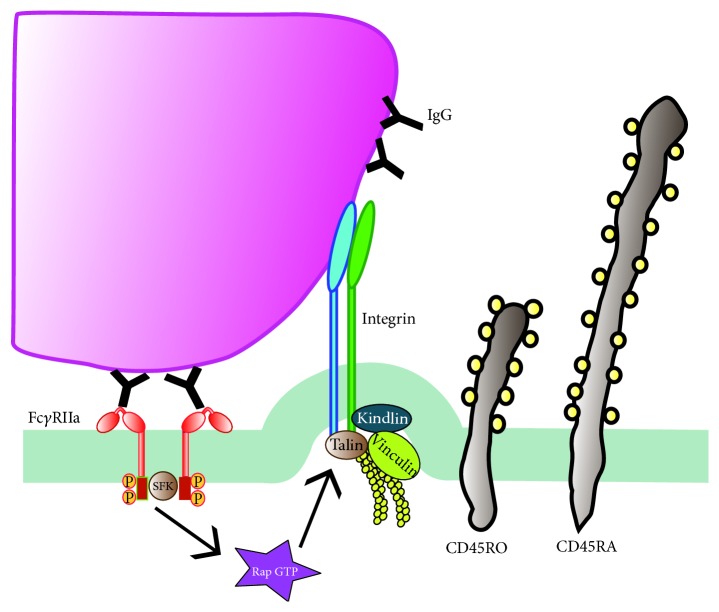
*Initial engagement of phagocytic receptors*. Aggregation Fc*γ*RIIa by an IgG opsonized particle initiates signaling. Receptor ITAMs (red rectangles) are phosphorylated by Src-family kinases (SFK) and recruit Syk. This leads to inside-out signaling for integrin (CR3) activation via the GTPase Rap. Activated integrin binds to adaptor molecules such as talin, vinculin, and kindlin-3 and connect to the actin cytoskeleton. Activated integrins also bind to the particle (via multiple possible ligands [113]) and form a diffusion barrier that excludes larger molecules, such as the transmembrane phosphatase CD45. This allows other Fc receptors to be engaged and increase the signaling for phagocytosis.

Besides its steric interference, there is another reason for removing CD45 from Fc*γ*Rs. The tyrosine phosphatase CD45 must be taken away from sites of Fc*γ*R engagement to allow full activation of Src tyrosine kinases, which phosphorylate ITAM sequences needed for activation of phagocytosis signaling [115]. First, CD45 must be allowed to diffuse more on the membrane. The lateral diffusion of CD45 is restricted by interactions between its cytoplasmic domain with ankyrin and spectrin molecules that connect to the actin cytoskeleton [118]. These interactions can be reduced by signals that alter the cytoskeleton and prime the cell for phagocytosis. TLR ligands, for example, LPS and bacterial DNA, can reduce the restricted diffusion of immunoreceptors [119]. Second, the more motile CD45 molecules need to be kept away from the engaged phagocytic receptor. This is achieved by the creation of a diffusion barrier made of activated integrins [114]. Fc*γ*Rs (and also G-protein coupled receptors or TLR) deliver signals for inside-out activation of integrins. Inactive integrins exist in a bent conformation that does not bind ligands. The signal from Fc*γ*R can produce DAG and Ca^2+^, which together activate CalDAG-GEF1 (a GEF for Rap). The small GTPase Rap in its GTP form is then able to recruit RIAM and talin to the cytoplasmic tail of the *β* subunit of integrins [120] (Figure 4(c)). This triggers the unfolding of the integrin into a high affinity “active” state. Kindlin-3 is another molecule that also binds to the *β* subunit of integrins causing their activation [121, 122]. The extended active integrin can then bind to many different ligands on the target particle [123]. Thus, integrins participate in Fc*γ*R-mediated phagocytosis by promoting adhesion to the opsonized particle [124]. In addition, the integrin molecules that get engaged by ligands get also tethered to the actin cytoskeleton and, with this, they form a diffusional barrier for CD45 molecules. The extended integrin bound to the target particle effectively pushes out the larger glycocalyx components, such as CD45 (Figure 5). As more integrin molecules get engaged they function as a progressive wave migrating ahead of the engaged Fc*γ*Rs, allowing new receptors to aggregate in microclusters [14] (Figure 5).

### 4.2. Actin Remodeling in Membrane Protrusions

**Figure 6:**
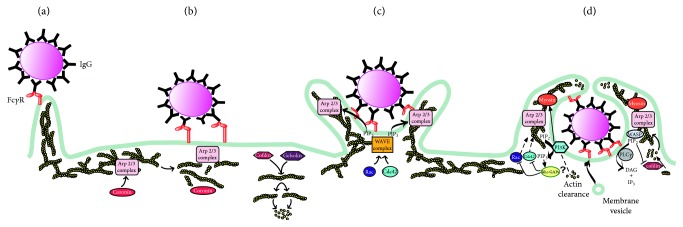
*Cytoskeleton changes during phagocytosis*. (a) Phagocytes explore their surroundings for phagocytic targets by projecting membrane ruffles, filopodia, and podosomes. These membranes contain mostly linear actin fibers. (b) Upon recognition of a target particle, the actin cytoskeleton is disrupted at the phagocytic cup by the action of coronins (F-actin debranching proteins) and cofilin and gelsolin (F-actin-severing proteins). (c) As more phagocytic receptors get engaged around the particle, the cell extends pseudopodia, which contain new branched actin fibers. Actin nucleation and F-actin polymerization are mediated by the Arp2/3 protein complex, which can be stimulated by the GTPases Rac and Cdc42, via the nucleation-promoting factor Scar/WAVE. (d) At the last step, depolymerization of actin filaments from the base of the nascent phagosome may facilitate curving of the membrane around the particle and provide room for fusion of internal vesicles, a source of endomembranes. Actin depolymerization is controlled by phosphatidylinositol 3-kinase (PI3K), through its product phosphatidylinositol (3,4,5)-trisphosphate (PIP_3_), which may recruit Rho GAPs that inactivate the GTPases Rac and Cdc42, thus reducing Arp2/3 activity. PIP_3_ also recruits myosins, which provide contractile activity that facilitates phagosome closure. At the same time, phospholipase C (PLC) cleaves phosphatidylinositol (4,5)-bisphosphate (PIP_2_) to generate diacylglycerol (DAG) and inositol-trisphosphate (IP_3_). The reduction of PIP_2_ will liberate cofilin and increase F-actin severing activity.

After a target particle is detected, the phagocytic process requires remodeling of the actin cytoskeleton to promote changes of the plasma membrane. The process is very complex and we have only a partial understanding of it. However, several important steps directed by actin remodeling, to form the pseudopodia that will cover the particle, can be identified. First, the membrane-associated cortical cytoskeleton, of the resting phagocyte, needs to be disrupted. Second, nucleation of actin filaments takes place in order to initiate F-actin polymerization and extension of pseudopodia. Third, actin gets depolymerized from the base of the phagocytic cup and the phagosome is closed at the distal end [13]. These steps of the precise temporal and spatial activation and inactivation of multiple proteins that govern F-actin dynamics are described next and presented in Figure 6.

A resting phagocyte presents a membrane-associated cortical cytoskeleton that provides cell shape. Upon activation, this cytoskeleton is disrupted by the action of coronins (F-actin debranching proteins) [125] and cofilin [126] and gelsolin [127] (F-actin-severing proteins). Coronin 1 rapidly accumulates at the nascent phagosome during both Fc*γ*R- and CR-mediated phagocytosis [125], and, in macrophages, it can interact with F-actin and inhibit the Arp2/3 complex [125]. Coronin 1 debranches F-actin leaving linear fibers that can be severed by cofilin and gelsolin (Figure 6, step (b)). Their activity is controlled by modulating their association with filaments, or by sequestering them away from filaments by binding to phosphoinositides, such as PI(4,5)P_2_ [127, 128]. In addition, the vesicular OCRL phosphatase activity to hydrolyze PI(4,5) P_2_ seems to contribute to the step of actin depolymerization [129]. The role for these enzymes in phagocytosis is much more complex than just described, and future research is needed in this area [13]. This initial disruption of the cytoskeleton has two consequences: it provides G-actin monomers for incorporation into new filaments and increases the mobility of nonligated receptors on the membrane (see previous section). The second step is the nucleation of actin filaments to initiate F-actin polymerization and extension of pseudopodia (Figure 6, step (c)). This is achieved mainly by the action of the Arp2/3 protein complex, which can be stimulated by different pathways. In fact, as indicated above the signaling pathways triggered by the best-studied phagocytic receptors, namely, Fc*γ*Rs and CRs, are very different (see Figures 1 and 3). For Fc*γ*R-mediated phagocytosis, Arp2/3 is recruited to the nascent phagocytic cup, where its actin-nucleating activity is stimulated by WASp and N-WASp [130, 131], which in turn are activated by Cdc42-GTP and PI(4,5)P_2_ [132]. In the case of CR-mediated phagocytosis, actin polymerization is associated with RhoA [133]. This GTPase recruits and stimulates mDia formins [108], which in turn also activate the Arp2/3 complex (Figure 3). However, other GTPases, such as Rap, seem to play a role in CR-mediated phagocytosis, independently of RhoA [134]. Rap-GTP also activates profilin, which is essential for actin polymerization via formins [135]. Rap can also activate the GTPase Rac [106]. But as discussed earlier, the role of Rac in complement-mediated phagocytosis remains a subject of debate.

### 4.3. Phagosome Sealing

The last step in phagosome formation is characterized by elimination of F-actin from the base of the phagocytic cup, just before the membrane protrusions fuse at the other end to seal the nascent phagosome (Figure 6, panel (d)). Depolymerization of actin filaments from the phagocytic cup may also facilitate curving of the membrane around the particle and provide room for fusion of internal vesicles, a source of endomembranes [129]. The mechanism for actin removal from the forming phagosome has been poorly defined, and much more research is needed in this topic. The mechanism for removing F-actin must include the termination of actin polymerization and the detachment and depolymerization of existing filaments. Both steps seem to be controlled by phosphoinositides, in particular PI(3,4,5)P_3_, the product of PI-3K. Inhibition of this enzyme prevents depolymerization of actin at the base of the phagocytic cup and arrests extension of pseudopods [136]. PI(3,4,5)P_3_ can activate Rho-family GAPs, which will induce deactivation of the GTPases stimulated during phagocytosis [137, 138]. Supporting this idea is the fact that PI-3K inhibition causes accumulation of activated Cdc42 and Rac at the phagocytic cup [92, 137]. However, because inhibition of PI-3K blocks phagocytosis even when GTPases are constitutively activated [137], this enzyme must control other molecules important for phagocytosis. One such molecule is PI(4,5)P_2_, which decreases by the action of PI-3K, but also by the action of PLC*γ*. Since PI(4,5)P_2_ sequesters cofilin and gelsolin and it is required for WASp activation, its reduction will increase F-actin severing (by liberation of cofilin and gelsolin) and reduce actin polymerization (by inhibition of WASp) [13]. Other molecules regulated by PI(3,4,5)P_3_ are myosins. Myosins exert contractile activity that functions as a purse string to facilitate phagosome closure [139–142] (Figure 6, step (d)).

Recently, the process of phagosome formation and closure has been revisited thanks to live microscopy with the technique of total internal reflection fluorescent microscopy (TIRFM) [143]. In this way, an important role for dynamin-2 in phagosome formation was revealed. Dynamin-2, which mediates the scission of endocytic vesicles, was recruited along with actin during phagosome formation, and depolymerization of actin led to impaired dynamin-2 recruitment or activity. Also, dynamin-2 accumulated at the site of phagosome closure [144]. Thus, it seems there is a cross-talk between actin and dynamin for phagosome formation and closure before dynamin functions for scission [144].

## 5. Phagolysosome Maturation

**Figure 7:**
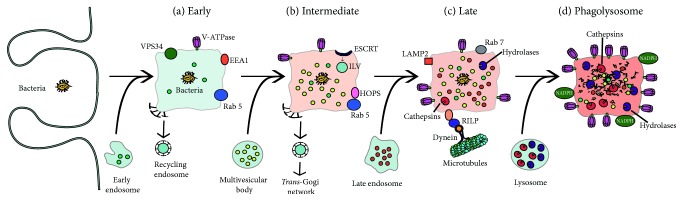
*Phagosome maturation*. (A) The nascent phagosome gets transformed into a microbicidal vacuole, the phagolysosome, by sequential interactions with vesicles from the endocytic pathway. Four stages of maturation have been described: early (a), intermediate (b), late (c), and phagolysosome (d). In this process, the phagosome becomes increasingly acidic by the action of a proton-pumping V-ATPase and gets various degradative enzymes. The composition of the membrane also changes to include molecules that control membrane fusion, such as the GTPases Rab. See text for details. EEA1, early endosome antigen 1; ESCRT, endosomal-sorting complex required for transport; HOPS, homotypic protein sorting; ILV, intraluminal vesicle; LAMP, lysosomal-associated membrane protein; NADPH, nicotinamide adenine dinucleotide phosphate oxidase; RILP, Rab-interacting lysosomal protein; vPS34, vacuolar protein-sorting 34.

The phagosome changes its membrane composition and its contents, to turn into a phagolysosome, a vesicle that can destroy the particle ingested. This transformation is known as phagosome maturation (Figure 7) and consists of successive fusion and fission interactions between the new phagosome and early endosomes, late endosomes, and finally lysosomes. At the end, the mature phagosome, also called phagolysosome, has a different membrane composition, which allows it to contain a very acidic and degradative environment [145, 146].

### 5.1. Early Phagosome

The new phagosome rapidly gets the properties of early endosomes, by fusing with sorting and recycling endosomes [28]. Its interior becomes a little acidic (pH 6.1–6.5) but it is not very destructive. Membrane fusion events between the phagosome and early endosomes are regulated by the small GTPase Rab5 [147, 148]. This membrane GTPase is required for the transition from an early to a late phagosome. Rab5 functions through the recruitment of EEA1 (early endosome antigen 1), which promotes fusion of the new phagosome with early endosomes [149]. Rab5 also recruits class III PI-3K human vacuolar protein-sorting 34 (hvPS34), which, in turn, generates phosphatidylinositol 3-phosphate [PI(3)P] [150]. This lipid then helps fix EEA1 to the cytosolic face of the phagosome and promotes recruitment of other proteins involved in phagosome maturation, including Rab7, a marker of late endosomes [151, 152]. EEA1 functions as a bridge that tethers early endosomes to incoming endocytic vesicles [153] and binds to syntaxin 13, a SNARE (soluble NSF-attachment protein receptor) protein required for membrane fusion [154]. Despite fusion with multiple early endosomes, the new phagosome does not seem to change size. This is due to the retrieval of vesicles to endosomes and the trans-Golgi network. Acidification of the phagosome lumen results from the gradual accumulation of active V-ATPases on the phagosome membrane. This V-ATPase is a multimeric protein complex that translocates protons (H^+^) into the lumen of the phagosome using cytosolic ATP as an energy source [155, 156] (Figure 7). In order to keep an electrical balance across the phagosome membrane, negative anions (mainly Cl^−^) also move inside, while cations (such as K^+^ and Na^+^) move outside [157, 158].

### 5.2. Intermediate Phagosome

As maturation proceeds, Rab5 is lost, and Rab7 appears on the membrane. The vpsC-homotypic protein-sorting (HOPS) complex mediates the transition from Rab5 to Rab7 endosomes [152] and may function in a similar fashion in phagosome maturation. Rab7 mediates the fusion of the phagosome with late endosomes [159]. At the same time, intraluminal vesicles are now formed. They contain membrane-associated molecules that are intended for degradation. These vesicles seem to arise from inwards budding and pinching of the limiting membrane of the phagosome [145]. The membrane proteins marked for degradation are ubiquitinated and associate with the endosomal-sorting complex required for transport (ESCRT) [160]. This complex forms a circular array that directs the vesicles into the lumen of the phagosome [161] (Figure 7).

### 5.3. Late Phagosome

Once the intermediate phagosome eliminates the proteins that will be recycled or degraded, it continues maturation to a late phagosome. Rab7 accumulates and becomes a marker for this stage. Rab7 recruits new proteins to the membrane. One such protein is Rab-interacting lysosomal protein (RILP), which binds to the dynein-dynactin complex [162, 163] and brings the phagosome in contact with microtubules. This mediates the centripetal movement of late phagosomes and lysosomes [162, 163] that brings the organelles in close contact so that SNARE proteins, such as VAMP (vesicle-associated membrane protein) 7 and VAMP8 can complete membrane fusion [164, 165]. At this stage, the lumen gets more acidic (pH 5.5–6.0), thanks to more V-ATPase molecules on the membrane [155] (Figure 7). In addition, lysosomal-associated membrane proteins (LAMPs) and luminal proteases (cathepsins and hydrolases) are incorporated from fusion with late endosomes or from the Golgi complex [145, 146].

### 5.4. Phagolysosome

The last stage in the maturation process involves fusion of late phagosomes with lysosomes, to become phagolysosomes. Phagolysosomes are the ultimate microbicidal organelle [28]. Phagolysosomes count with many sophisticated mechanisms directed to eliminate and degrade microorganisms. They are highly acidic (pH as low as 4.5) thanks to the large number of V-ATPase molecules on their membrane [156]. Phagolysosomes are also characterized by a PI(3)P-enriched internal membrane [166, 167] and by the lack of mannose-6-phosphate receptors [168]. They also contain a number of hydrolytic enzymes, including various cathepsins, proteases, lysozymes, and lipases [155]. Other microbicidal components of the phagosome are scavenger molecules, such as lactoferrin that sequesters the iron required by some bacteria [169] and the NADPH oxidase that generates superoxide (O_2_^−^) [170] (Figure 7). Superoxide can dismutate to H_2_O_2_, which can in turn react with O_2_^−^ to generate more-complex reactive oxygen species (ROS), such as hydroxyl radicals and singlet oxygen [171]. In addition, H_2_O_2_ can be combined with Cl^−^ ions into hypochlorous acid by the enzyme myeloperoxidase [172].

## 6. Conclusion

Phagocytosis is an elegant and very complex process for the ingestion and elimination of pathogens and apoptotic cells. It is performed by a series of cells we call professional phagocytes. They are monocytes, macrophages, neutrophils, dendritic cells, osteoclasts, and eosinophils. It is evident that phagocytosis is fundamental for tissue homeostasis, controlling important aspects of inflammation and the immune response. Clearly, the many cell types that can perform phagocytosis and the overwhelming number of different phagocytic targets require more than one mechanism to complete this cellular function. We have presented the main four steps of phagocytosis to provide a general view of the whole process. Still, we have to keep in mind that this description corresponds primarily to opsonic receptors. We have very little knowledge of the signaling pathways other phagocytic receptors activate. Similarly, the process of phagosome maturation has gained much information from studies on vesicular traffic. Yet, important gaps remain in every step. Also, how the final phagolysosome completes its antimicrobial or degradative functions is not completely clear. But, the fact that several microbial pathogens have developed special ways for interfering with phagolysosome function gives us another opportunity to learn from them novel aspects on phagocytosis. In addition, the resolution of the phagolysosome, after the infection or the inflammation processes have terminated, is an area that has brought very little attention. What are the molecular details and functional implications of ingesting different particles? How the various phagocytic receptors on the same phagocyte cooperate? And how the various phagocytes participate in tissue homeostasis? These are important questions that future research in this exciting area will have to address. An improved understanding of phagocytosis is essential for the clear implications it has for antigen presentation and autoimmune disease.

## Figures and Tables

**Table 1:** Human phagocytic receptors and their ligands.

Receptor	Ligands	Reference(s)
*Pattern-recognition receptors*		
Dectin-1	Polysaccharides of some yeast cells	[29]
Mannose receptor	Mannan	[30]
CD14	Lipopolysaccharide-binding protein	[31]
Scavenger receptor A	Lipopolysaccharide, lipoteichoic acid	[32, 33]
CD36	*Plasmodium falciparum*-infected erythrocytes	[40]
MARCO	Bacteria	[41]
*Opsonic receptors*		
Fc*γ*RI (CD64)	IgG1 = IgG3 > IgG4	[42]
Fc*γ*RIIa (CD32a)	IgG3 ≥ IgG1 = IgG2	[42]
Fc*γ*RIIIa (CD16a)	IgG	[42]
Fc*α*RI (CD89)	IgA1, IgA2	[11, 43]
Fc*ε*RI	IgE	[44]
CR1 (CD35)	Mannan-binding lectin, C1q, C4b, C3b	[45]
CR3 (*α*_M_*β*2, CD11b/CD18, Mac-1)	iC3b	[46]
CR4 (*α*_V_*β*2, CD11c/CD18, gp190/95)	iC3b	[46]
*α*5*β*1	Fibronectin, vitronectin	[47]
*Apoptotic body receptors*		
TIM-1^*∗*^	Phosphatidylserine	[48]
TIM-4^*∗*^	Phosphatidylserine	[48]
Stabilin-2	Phosphatidylserine	[49]
BAI-1^*∗*^	Phosphatidylserine	[50]
*α*_V_ *β*3	MFG-E8^*∗*^	[51]
*α*_V_ *β*5	Apoptotic cells	[52]
CD36	Oxidized lipids	[53]

^*∗*^TIM, T cell immunoglobulin mucin; BAI-1, brain-specific angiogenesis inhibitor 1; MFG, milk fat globule.
